# The development of an online decision aid to support persons having a genetic predisposition to cancer and their partners during reproductive decision-making: a usability and pilot study

**DOI:** 10.1007/s10689-018-0092-4

**Published:** 2018-05-30

**Authors:** Kelly Reumkens, Marly H. E. Tummers, Joyce J. G. Gietel-Habets, Sander M. J. van Kuijk, Cora M. Aalfs, Christi J. van Asperen, Margreet G. E. M. Ausems, Margriet Collée, Charlotte J. Dommering, C. Marleen Kets, Lizet E. van der Kolk, Jan C. Oosterwijk, Vivianne C. G. Tjan-Heijnen, Trudy van der Weijden, Christine E. M. de Die-Smulders, Liesbeth A. D. M. van Osch

**Affiliations:** 1 Department of Clinical Genetics, Maastricht University Medical Centre+, Postbox 5800, 6202 AZ Maastricht, The Netherlands; 2 GROW School for Oncology and Developmental Biology, Maastricht University Medical Centre+, Maastricht, The Netherlands; 3 Department of Clinical Epidemiology and Medical Technology Assessment, Maastricht University Medical Centre+, Maastricht, The Netherlands; 4 Department of Clinical Genetics, Academic Medical Centre, Amsterdam, The Netherlands; 5 Department of Clinical Genetics, Leiden University Medical Center, Leiden, The Netherlands; 6 Division of Biomedical Genetics, Department of Genetics, University Medical Center Utrecht, Utrecht, the Netherlands; 7 Department of Clinical Genetics, Erasmus University Medical Center, Rotterdam, The Netherlands; 8 Department of Clinical Genetics, VU University Hospital, Amsterdam, The Netherlands; 9 Department of Human Genetics, Radboud University Medical Center Nijmegen, Nijmegen, the Netherlands; 10 Family Cancer Clinic, Netherlands Cancer Institute, Amsterdam, The Netherlands; 11 Department of Genetics, Groningen University Medical Center, University of Groningen, Groningen, the Netherlands; 12 Department of Medical Oncology, Maastricht University Medical Centre+, Maastricht, The Netherlands; 13 School CAPHRI Care and Public Health Research Institute, Maastricht University Medical Centre+, Maastricht, The Netherlands; 14 Department of Family Medicine, Maastricht University Medical Centre+, Maastricht, The Netherlands; 15 Department of Health Promotion, Maastricht University Medical Centre+, Maastricht, The Netherlands

**Keywords:** Reproductive decision-making, Counselling, Decision aid, Hereditary cancer, Informed decision-making, Oncology, Patient education

## Abstract

An online decision aid to support persons having a genetic predisposition to cancer and their partners during reproductive decision-making was developed. A two-phase usability test was conducted among 12 couples (N = 22; 2 persons participated without their partner) at risk for hereditary cancer and 15 health care providers. Couples and health care providers expressed similar suggestions for improvements, and evaluated the modified decision aid as acceptable, easy to use, and comprehensible. The final decision aid was pilot tested (N = 16) with paired sample t tests comparing main outcomes (decisional conflict, knowledge, realistic expectations regarding the reproductive options and decision self-efficacy) before (T0), immediately (T1) and 2 weeks after (T2) use of the decision aid. Pilot testing indicated decreased decisional conflict scores, increased knowledge, and improved realistic expectations regarding the reproductive options, at T1 and T2. No effect was found for couples’ decision self-efficacy. The positive findings during usability testing were thus reflected in the pilot study. The decision aid will be further evaluated in a nationwide pretest–posttest study to facilitate implementation in the onco-genetic counselling setting. Ultimately, it is expected that the decision aid will enable end-users to make an informed decision.

## Introduction

A predisposition for hereditary cancer is usually autosomal dominant, implying that there is a 50% risk of transmitting the mutation to offspring. Transmission of a predisposition for hereditary cancer to offspring means passing on a generally highly increased risk of developing cancer. For the relatively frequent breast cancer gene mutations in *BRCA1* or *BRCA2* this implies risks of 27–57% and 6–40% of developing breast respectively ovarian cancer by the age of 70 [1, 2]. This knowledge may evoke challenging reproductive decision-making processes among persons having a genetic predisposition to cancer and their partners [3–6]. Couples with a predisposition for hereditary cancer who want a genetically related child can opt for natural conception without genetic testing, accepting the risk of passing on the predisposition for cancer to a child, or they could opt for prenatal diagnosis (PND) assuming the intention to terminate the pregnancy if the fetus has the mutation [7], or for preimplantation genetic diagnosis (PGD), to prevent transmission of the mutation to their offspring. PGD involves a multi-stage diagnostic process in which embryos are examined for the presence of the familial mutation before pregnancy is established [7]. Annually approximately 60 couples with a predisposition for hereditary cancer start a PGD procedure in the Netherlands [8], a number that has been steadily increasing since its introduction 10 years ago. In 2016, a total of 40 couples with a predisposition for hereditary breast and ovarian cancer (*HBOC*) started a PGD procedure and seven couples with familial adenomatous polyposis (*FAP*). The uptake of PND for hereditary cancer is relatively low (< 2%) with up till 2013 only a total of six couples with *HBOC* performed PND (< 0.2%) and a total of six couples with *FAP* (1.6%) [9]. Although awareness of PGD (66%) and PND (61%) are similar, the acceptability of PGD (80%) is notably higher compared to PND (26%) [10].

The decision regarding which reproductive option to choose is highly preference-sensitive. Research has shown that persons having a genetic predisposition to cancer and their partners may experience the reproductive decision-making process as complex [6, 11–13]. A recent study among couples with *HBOC*, showed that 43% experienced reproductive decision-making as (very) difficult [13]. Feelings of uncertainty and guilt may be experienced, particularly among couples who opt for natural conception without genetic testing [5]. In deliberating the reproductive options, couples consider personal values and (dis)advantages of all options, which can be categorized into physical (e.g. physical burden of IVF treatment necessary for PGD), psychological (e.g. loss of romance regarding pregnancy), social (e.g. eliminate mutation in family line), ethical (e.g. moral duty to protect the child) and practical considerations (e.g. frequent hospital appointments) [5].

The decision regarding which reproductive option to pursue should ideally involve an informed decision-making process by an educated and empowered couple. In order to promote informed decision-making, decisional support strategies can be effective. Compared to usual care interventions, decision aids have been found to improve people’s knowledge regarding their options, reduce decisional conflict, and decrease the proportion of people remaining undecided [13–16]. Incorporating a patient decision aid in reproductive counselling can therefore be helpful in supporting persons having a genetic predisposition to cancer and their partners in making their reproductive decision [5, 17].

The present report is part of a project on the development and implementation of an online decision aid. Firstly, we conducted a needs assessment study regarding the preferences and needs of couples and health care providers regarding the decision aid [18]. We integrated the results of the needs assessment study with knowledge on reproductive decisional motives and considerations and designed a concept version of the decision aid [18]. Its user friendliness, strengths and limitations were assessed during usability testing and some modifications were made. Subsequently, the final decision aid was pilot tested. We report on the results of the usability testing and the preliminary results regarding the effectiveness of the decision aid, generates during beta testing (i.e. end-user testing) by means of a pilot study [19].

## Methods

### Developmental process and content of the decision aid

The decision aid was developed according to the International Patient Decision Aids Standards [20] in collaboration with a steering group including health care providers (e.g. clinical geneticists, and social workers), experts in health communication and medical decision-making, psychologists and persons having a genetic predisposition to cancer and their partners who are planning to have children. Our needs assessment study showed many similarities between the expressed preferences and needs of both couples and health care providers concerning the content, barriers and facilitating factors regarding the use of the decision aid, and its implementation [18]. The prototype of the decision aid contained:

1. Information about the risk of transmitting the mutation to offspring.
2. Information about couples’ options to have genetically related child(ren) (natural conception without genetic testing, PND and PGD) with the aim to increase knowledge. In the needs assessment study, participants agreed upon natural conception without genetic testing, PND and PGD as main reproductive options to be included in the decision aid [18]. Adoption and use of donor gametes are mentioned in the decision aid to make couples aware of the existence of these options. However, as most couples pursue their wish to have genetically related child(ren) [21], these options were not included further.
3. Probabilities of different outcomes and the burden of the treatment of reproductive options (e.g. risk of miscarriage after PND, likelihood of pregnancy with PGD) to increase participants’ accuracy of risk comprehension. Based on current recommendations [22] and the preferences of end-users, probabilities were presented in multiple suitable formats using text and videos (e.g. verbal, and population diagrams).
4. A summary table of important features of each reproductive option to facilitate comparison.
5. Values clarification exercises (VCE) [23]. Participants were presented with 18 statements representing values and motives considered important for reproductive decision-making [5]. Participants were asked to rate personal agreement of each statement on a scale from 1 (disagree) to 6 (agree). By linking login codes, an automated combined overview of both partners’ input could be generated to facilitate communication about agreements and possible discrepancies.
6. A question prompt sheet, providing examples of questions and requests for additional information and space for own questions, to facilitate discussion with health professionals and others.
7. Information regarding the scientific resources used to underpin the decision aid content, information on the development team (including ‘conflicts of interest COI’), funding resources and contact information.

### Usability testing

Minor textual revisions were made after review of the prototype decision aid by the steering group. Subsequently, a two-phase usability test with couples and health care providers was conducted. After the first phase the decision aid was adapted based on provided feedback. To make final modifications, a second usability phase was conducted.

#### Participants and recruitment

Dutch couples who consider PGD are referred to the Clinical Genetics Department of the Maastricht University Medical Centre (Maastricht UMC+) for an once only informative consultation. The couples are registered in a database. Eligible participants for the usability test were selected from this database. Couples were eligible for participation if one partner had a confirmed mutation for a hereditary cancer syndrome for which PND and PGD are available in the Netherlands, if they had made a reproductive decision (as indicated in medical records), if both partners were 18 years or older, had sufficient knowledge of the Dutch language, and if they had ample experience with the use of computers and the Internet. Although participation of both partners was encouraged, participation of one partner (regardless of being carrier) was allowed. Couples who provided written informed consent for participation were contacted by telephone to schedule an appointment at the hospital, or, if preferred, at the couple’s home.

Furthermore, two representatives of each of the nine Clinical Genetics Departments in the Netherlands, who were directly involved in the counseling and care of end-users (e.g. clinical geneticists and gynecological oncologists) were invited to participate (N = 18). Appointments were scheduled at convenient workplaces.

#### Procedures

Usability testing was conducted using a mixed methods design (i.e. qualitative and quantitative) in between March and June 2016. First, couples and health care providers were asked to fill in a brief questionnaire. Age, gender, educational level, carrier status, type of cancer, and experience with the use of computers and the Internet were assessed for couples. Health care providers completed questions concerning their professional perspectives regarding the feasibility of implementing the decision aid. Subsequently, participants were invited to an online ‘cognitive walkthrough’ of the decision aid in presence of the researcher. They were asked to think aloud and to express freely their opinion regarding its content, functionalities, format and layout [24]. The researchers occasionally asked predetermined questions. ‘Thinking aloud’ sessions were followed by interviews. The content of the semi-structured topic guides for the interviews focused on the perceived comprehensibility, usability, efficiency and acceptability of the decision aid and were largely similar for the couples and the health care providers. At the end of the interviews, couples were asked to evaluate the decision aid with regard to content, lay-out and usability on a scale of 1–10 and to complete the System Usability Scale (SUS) for the subjective assessment of usability [25–27]. Couples received 15 euros in vouchers and their travel expenses were reimbursed. Health care providers were asked to evaluate the decision aid in terms of quality, completeness, lay-out and usability on a scale of 1–10 with higher scores indicating better usability.

#### Data analysis

Qualitative data derived from the audiotaped ‘thinking aloud’ sessions and the interviews were transcribed verbatim. Open coding (developing categories of information) and axial coding (exploring the relationship of categories) was performed independently by two researchers (K.R. and M.T.) to derive and categorize main issues. Data from the demographic questionnaire and SUS were analysed by means of descriptive statistics and quantified as mean and standard deviation or absolute number and percentage, using SPSS version 23.

### Pilot study

#### Participants and recruitment

Health care providers of all Clinical Genetics Departments in the Netherlands recruited eligible couples during or after oncogenetic consultation. The same inclusion criteria were used as for usability testing, except that couples had not yet made a reproductive decision. Couples were eligible if they expressed the wish to have children within 5 years and have not yet made a definitive decision regarding their preferred reproductive option. Although participation of both partners was encouraged, participation of one partner was allowed.

#### Procedures and instrumentation

Adaptation of feedback provided during usability testing resulted in the final decision aid. It was pilot tested from November 2016 to January 2017. Eligible couples were provided with an information brochure, to introduce the study together with a link to an online registration page. After registration, participants received online information about the study and an online informed consent form. After providing consent, participants were directed to an online (baseline) questionnaire (T0) and received a personal login code for the decision aid. Duration of visits and page visits were monitored. Immediately after use of the decision aid, participants were directed to the second online questionnaire (T1). Two weeks after baseline, participants were asked by e-mail to complete the last questionnaire (T2). Participants who did not complete the questionnaire received a reminder by e-mail. Questionnaires were completed separately by both partners. After completing all questionnaires, participants received 15 euros in vouchers.

A demographic questionnaire assessed gender, age, educational level, carrier status, type of cancer, and personal history of cancer at T0. Less than primary, primary and lower secondary education was considered as low education levels. Upper secondary and post-secondary non-tertiary education was considered as middle education levels. Tertiary education was considered as a high education level. In addition, participants’ reproductive history was assessed. The primary outcome measure, i.e. participants’ level of *decisional conflict*, was assessed by the Decisional Conflict Scale at T0, T1 and T2. The questionnaire contains 16 items, each scored on a 5-point Likert scale ranging from 0 (strongly agree) to 4 (strongly disagree). Total scores range from 0 (no decisional conflict) to 100 (extremely high decisional conflict) [28]. Participants’ current *knowledge of the three reproductive options* was assessed by 15 closed-ended questions (true/false/not sure) at T0, T1 and T2, see Table 1 [10]. Three questions measured participants’ knowledge of natural conception without genetic testing (e.g. ‘when opting for natural conception, there is a 50% risk of transmitting the mutation to offspring’), five questions measured knowledge of PND (e.g. ‘prenatal diagnosis takes place during pregnancy’) and seven questions measured knowledge of PGD (e.g. ‘In vitro fertilization (IVF) is necessary to perform PGD’). One point was provided for each correctly answered question, which could lead to a maximum score of 15. *Realistic expectations regarding the three reproductive options* were assessed by three questions at T0, T1 and T2 (i.e. “what is the extra risk of miscarriage due to PND?”, “what is the chance of pregnancy after one IVF treatment with PGD?”, “what is the risk of complications with PGD?”). These questions contained 8–11 response options. One point was provided for each correctly answered question, which could lead to a maximum score of three [29]. Participants’ *decision self-efficacy* was assessed by the Decision Self-Efficacy Scale at T0, T1 and T2. The questionnaire contains 11 items, each scored on a 5-point Likert scale ranging from 0 (not at all confident) to 4 (very confident). Total scores range from 0 (extremely low self-efficacy) to 100 (extremely high self-efficacy) [30].

**Table 1:** Knowledge of the three reproductive options (pilot study)

No.	Item
	Natural conception without genetic testing
1	When opting for natural conception, there is a 50% risk of transmitting the mutation to offspring (T)
2	When opting for natural conception, besides standard procedures, there will be no extra examinations performed during pregnancy (T)
3	When opting for natural conception, during delivery it is already clear whether your child has the mutation (F)
	Prenatal diagnosis
4	Prenatal diagnosis takes place during pregnancy (T)
5	When opting for prenatal diagnosis, you and your partner can naturally conceive (T)
6	Results of prenatal diagnosis will always follow within 1 week (F)
7	Prenatal diagnosis is possible from 6 weeks of pregnancy upon (F)
8	Prenatal diagnosis is possible in most of the medical centers in the Netherlands (T)
	PGD
9	In vitro fertilization (IVF) is necessary to perform PGD (T)
10	PGD is possible in every hospital in the Netherlands (F)
11	For PGD, cooperation of family members is a prerequisite (T)
12	Hormone-use by the woman is necessary for a PGD treatment (T)
13	PGD takes place before the woman is pregnant (T)
14	A PGD treatment takes at least 6 months (T)
15	In the Netherlands, a woman’s maximum age for PGD is 45 (F)

Total score range 0–15

#### Data analysis

To test for intra-couple correlation, we compared two models for testing the difference in the main outcome (decisional conflict); one linear mixed-effects model in which clustering within couples was corrected for, and one model without correction. Both models yielded similar results, and a likelihood-ratio test showed that correction did not lead to a better fit (likelihood ratio = 0.60, p = 0.44). Therefore, all participants can be analyzed as separate individuals and we chose to report the simpler model without correction for clustering and used paired sample t tests to compute differences between the first and subsequent measurements. P-values of < 0.05 were considered to indicate statistical significance.

### Compliance with ethical standards

This study was approved by the medical ethics committee of Maastricht UMC+ (METC 14-5-089) and registered in the Dutch Trial Register (NTR5467). The authors declare that they have no conflict of interest. All procedures performed in this study were in accordance with the ethical standards of the medical ethics committee of Maastricht University Medical Centre and have been performed in accordance with the ethical standards as laid down in the 1964 Declaration of Helsinki and its later amendments. Informed consent was obtained from all patients included in this study.

## Results

### Usability testing

#### Couples’ characteristics

Thirty-nine couples who had reproductive counseling for hereditary cancer between 2013 and 2015, were invited for participation by mail. Twelve couples provided written informed consent (N = 22; 2 persons participated without their partner), and participated in usability testing (response rate 30.8%). Main reasons for non-participation were a lack of time and not wanting to relive the psychological burden associated with reproductive decision-making. The mean age was 34.5 years for males (SD = 4.5) and 29.9 years (SD = 3.7) for females. Most participants were highly educated (64%), a minority had lower education levels (14%) and 23% had an average education level. The types of cancer concerned hereditary breast and ovarian cancer (N = 6), familial adenomatous polyposis (N = 2), Lynch syndrome (N = 3) and multiple endocrine neoplasia (N = 1).

#### Usability results couples

During the first phase of the usability test, three couples and one female participant without partner (N = 7 persons) participated. A frequently expressed concern during the ‘think aloud’ sessions was the amount of scrolling needed to read all provided information. Other suggestions mainly pertained to textual improvements and adaptations to further improve lay-out (e.g. change the order of information and a change of colors). The average duration of the sessions was 71 min (range 60–80). Seven couples and one female participant without partner (N = 15 persons) participated in the second phase of usability testing to make final modifications. In general, couples appreciated the lay-out and stated that information in the decision aid was clear and comprehensible. Most couples indicated that they would have used the decision aid, if it had been available at the time of reproductive decision-making, and stated that all functions included in the decision aid were well integrated (Table 2). Couples generally agreed that the decision aid was not unnecessarily complex (Table 2). With a mean SUS score (range 0–100) of 91.33 (SD = 7.61), the decision aid’s usability was considered high. Couples graded the decision aid on a scale of 1–10 with a mean of 8.5 (SD = 0.5) for the content, a mean of 8.3 (SD = 0.8) for lay-out and a mean of 8.0 (SD = 0.9) for usability.

**Table 2:** System usability scale results (usability study)

No.	Item	N = 15 Mean (SD)
1	I think that I would like to use the DA frequently	3.07 (1.16)
2	I found the DA unnecessarily complex	0.20 (0.78)
3	I thought the DA was easy to use	3.53 (0.74)
4	I think that I would need technical support to be able to use the DA	0.00 (0.00)
5	I found the various functions included in the DA well integrated	3.47 (0.64)
6	I thought there was too much inconsistency in the DA	0.40 (1.06)
7	I would imagine that people would learn to use the DA very quickly	3.53 (0.52)
8	I found the DA very cumbersome to use	0.07 (0.26)
9	I felt very confident using the DA	3.73 (0.46)
10	I needed to learn a lot before I could get going with the DA	0.13 (0.35)
Total score		91.33 (7.61)

Total score range 0–100; higher scores indicate higher perceived usability

*DA* decision aid

#### Health care providers’ characteristics

Fifteen health care providers participated (response rate 83.3%) in the ‘think aloud’ sessions and individual interviews (1 male and 14 females). The mean age for males was 61 years and mean age for females was 43.5 years (SD = 6.7). These health care providers were clinical geneticists (N = 6), gynecological oncologists (N = 3), genetic counselors (N = 2), social worker (N = 1), medical oncologists (N = 2) and an ophthalmologist (N = 1; involved in the care and counseling of persons with a predisposition for retinoblastoma and their partners). The majority had more than 5 years of work experience in the area of oncogenetic counseling and 80% had no or limited experience with the use of decision aids.

#### Usability results health care providers

The average duration of the sessions was 59 min (range 15–80). Similar to couples, health care providers highly appreciated the lay-out of the decision aid, although during ‘think aloud’ sessions the use of more subtle colors was suggested. Several textual suggestions (e.g. avoidance of medical/technical terms) and suggestions to change the order of information were provided. The different forms of information provision (e.g. written and video-based) and the (VCE) were appreciated. To promote (continued) implementation, it was suggested to include a link to the decision aid in the standard report couples receive after consultation. The format of the final decision aid was considered as acceptable, easy to use and comprehensible. Health care providers graded the decision aid on a scale of 1–10 with a mean score of 8.2 (SD = 0.5) for quality, a mean of 8.5 (SD = 0.5) for completeness, a mean of 7.9 (SD = 0.4) for lay-out and a mean of 7.3 (SD = 0.7) for usability. The moderate mean usability score of 7.3 was likely due to the order in which information was presented in the decision aid. Changes were made to the decision aid based on feedback provided.

### Pilot study

#### Participants’ characteristics

Eight couples (N = 16) participated in the pilot study. A response rate could not be estimated because the exact number of patients invited by each Clinical Genetics Department is unknown due to the large number of counselors recruiting. Table 3 shows an overview of the sample characteristics. Participants’ average age was 32.4 years for males (SD = 4.6) and 29.1 years (SD = 4.3) for females. None of the participants had a low education level. Of the participants, 68.8% already had a preferred option in mind at baseline, 31.2% did not. None of the participants changed their mind from T0–T1 and from T0–T2. The mean time spent using the decision aid was 27 min (range 2–104 min) and participants viewed on average 20 out of 36 pages. 69% viewed at least 25 pages with pages on contact information and disclaimer being the least viewed.

**Table 3:** Sample characteristics (pilot study)

Sample characteristics (n = 16)	N	%
Gender		
Male (M)	8	50.0
Female (F)	8	50.0
Age (years)		
Male	32.4 (SD = 4.6)	
Female	29.1 (SD = 4.3)	
Education		
Middle	7	43.8
High	9	56.2
Carrier status		
Male	2	12.5
Female	6	87.5
Syndrome		
Hereditary breast and ovarian cancer	7 (5F/2M)	87.5
Lynch syndrome	1 (F)	12.5
Have (had) cancer		
Yes	4	25.0
No	12	75.0
Reproductive history		
Children		
Yes	2	12.5
No	14	87.5
Planning to have children		
Currently pregnant	2	12.5
Planning to have children within 5 years	14	87.5
Preferred reproductive option in mind at T0		
Yes	11	68.8
No	5	31.2

#### Preliminary effects of decision aid

All 16 participants completed T0 and T1 and 15 participants completed T2. As shown in Table 4, mean decisional conflict scores (range 0–100) decreased from a mean of 27.6 (SD = 19.3) at baseline, to 11.8 (SD = 15.3) at T1 (t = 5.73; p < 0.001; ES = 1.73) and 8.3 (SD = 6.4) at T2 (t = 3.37; p = 0.01; ES = 1.12). The mean level of knowledge (range 0–15) increased from 8.2 (SD = 3.5) at baseline, to 12.4 (SD = 3.7) at T1 (t = − 7.73; p < 0.001; ES = − 1.93) and 12.8 (SD = 2.1) at T2 (t = − 10.05; p < 0.001; ES = − 2.69). Further, realistic expectations regarding the three reproductive options (range 0–3) increased from 0.4 (SD = 0.5) at baseline, to 1.9 (SD = 1.0) at T1 (t = − 6.45; p < 0.001; ES = − 0.75) and 1.3 (SD = 1.0) at T2 (t = − 4.33; p < 0.001; ES = − 0.25). Couples’ decision self-efficacy (range 0–100) slightly increased but did not significantly change over time from 80.4 (SD = 16.9) at baseline, to 81.3 (SD = 16.0) at T1 (t = − 0.28; p = 0.782; ES = − 0.07) and 83.9 (SD = 19.6) at T2 (t = − 0.38; p = 0.708; ES = − 0.10).

**Table 4:** Overview of main outcome measures (pilot study)

Questionnaire (N = 16)	Means (SD)			Paired samples t-test			
	T0	T1	T2	T0–T1		T0–T2	
				*T*	*p*	*T*	*p*
Decisional conflict							
Total score (0–100)	27.6 (19.3)	11.8 (15.3)	8.3 (6.4)	5.73	< 0.001	3.37	0.010
Uncertainty	43.8 (28.5)	27.6 (30.4)	29.2 (27.7)	3.67	0.002	2.19	0.047
Informed	40.6 (22.5)	17.7 (18.5)	11.3 (13.7)	5.21	< 0.001	5.97	< 0.001
Values clarity	30.2 (26.2)	19.3 (21.9)	11.9 (15.6)	2.78	0.014	3.54	0.004
Support	24.0 (16.6)	17.7 (18.0)	13.7 (14.1)	1.60	0.131	2.65	0.020
Effective decision	17.6 (15.0)	8.0 (15.3)	5.6 (11.5)	4.54	< 0.001	1.71	0.126
Knowledge							
Total score (0–15)	8.2 (3.5)	12.4 (3.7)	12.8 (2.1)	− 7.73	<0.001	− 10.05	< 0.001
Natural conception (0–3)	2.0 (1.0)	2.3 (0.9)	2.5 (0.9)	− 1.78	0.096	− 2.45	0.028
PND (0–5)	1.8 (1.7)	3.9 (1.4)	4.5 (2.0)	− 5.33	< 0.001	− 3.96	< 0.001
PGD (0–7)	4.4 (1.8)	6.1 (1.9)	6.4 (0.9)	− 5.65	< 0.001	− 3.67	0.003
Realistic expectations (0–3)	0.4 (0.5)	1.9 (1.0)	1.3 (1.0)	− 6.45	< 0.001	− 4.33	< 0.001
Decision self-efficacy (0–100)	80.4 (16.9)	81.3 (16.0)	83.9 (19.6)	− 0.28	0.782	− 0.38	0.708

## Discussion

This study presents the development and preliminary evaluation of an online decision aid that aims to support persons having a genetic predisposition to cancer and their partners during their reproductive decision-making. Previous studies demonstrated that these couples may experience the decision-making process as complex [6, 11, 12, 31]. The decision aid aims to decrease participants’ level of decisional conflict, increase participants’ knowledge, improve realistic expectations regarding the available reproductive options and increase participants’ decision self-efficacy. The high mean score on the SUS indicates that the decision aid meets the needs of the target population. During usability testing, couples and health care providers expressed similar suggestions for improvements. Overall the decision aid was evaluated as acceptable, easy to use, and comprehensible. The positive findings during usability testing were reflected in the preliminary results regarding efficacy of the decision aid, indicated by reduction of couples’ decisional conflict levels, increases in knowledge levels and improvement of realistic expectations regarding available reproductive options. This suggests that with use of the decision aid, informed decision-making among persons having a genetic predisposition to cancer and their partners during reproductive decision-making may be improved. Despite the complexity of the decision, couples’ confidence in their ability to make a decision was already high at baseline and did not increase as a result of decision aid use, possibly reflecting a ceiling effect. This may be explained by the finding that 11 out of 16 participants already had a preferred reproductive option in mind at baseline, indicating that couples had already considered the available reproductive options to a certain extent. However, although couples overall felt confident about making a reproductive decision, baseline knowledge levels were relatively low. As a solid knowledge base is regarded as a prerequisite for informed decision-making [32], this finding further emphasizes the need for informational support among our sample.

In order to optimize the impact of the decision aid with regard to decision self-efficacy, the use of modeling techniques may be considered [33], for instance by means of incorporating narrative stories in the decision aid. Previous research, including a needs assessment regarding the current decision aid, indicated that both couples and health care providers advocate the provision of narrative stories during reproductive decision-making [5, 18]. These personal stories detail the experiences of couples with reproductive decision-making and are aimed at providing illustrative examples of others’ experiences. Narratives can be useful in overcoming preconceived beliefs and cognitive biases and integrating narratives into healthcare communication is increasingly being recommended [34, 35]. Currently insufficient evidence exists about the effectiveness of narrative stories on informed decision-making and how to incorporate these stories in decision support tools [36, 37]. Future research should explore essential elements of the content of narrative stories and their effectiveness in facilitating decision-making.

A limitation of this study relates to the small sample size and selection bias towards higher educated and possibly higher health literate users which may limit generalizability of the results. Subsequently, the decision aid will be further evaluated in a nation-wide effect study to draw more robust conclusions. Ultimately, it is expected that the decision aid will enable end-users to make an informed decision, which may lessen the negative psychological impact of decision-making on couples’ daily life and wellbeing.

## Conclusions

The current findings indicate that the decision aid was well received by both couples and health care providers as reflected in high usability scores and promising preliminary efficacy during the pilot study. The decision aid will be further evaluated in a nationwide pretest–posttest study.
